# Notes and News

**Published:** 1904-12-24

**Authors:** 


### Notes and News.

A sanatorium for consumptives has been opened at Leghorn as a memorial to King Humbert of Italy.

The Local Government Board Vaccination Grant has been awarded to Mr. John Campbell, M.R.C.S., L.R.C.P., Public Vaccinator, No. 3 District, Goole Union, Yorks.

The medical student who was reported to have died of plague at Oporto in reality died of blood-poisoning contracted through wounding himself whilst dissecting.

To disseminate useful knowledge in relation to the prevention of consumption a museum of tuberculosis has been opened at Karlsruhe after the model of the one in Berlin.

The foundation-stone of an English hospital to be conducted by the Nursing Sisters of the Little Company of Mary has been laid in Rome. Cardinal Respighi presided over the ceremonies.

It is unfortunate that Siberian plague has broken out in the fur factories of two districts where fur coats for the Russian Army are being manufactured. The delivery of the fur coats is being suspended.

Lord Strathcona and Mount Royal has arranged to preside at a festival dinner at the Whitehall Rooms, in aid of the National Hospital for the Paralysed and Epileptic, on April 13 next.

Through the will of the late Mr. Henry Bloom Noble, of Douglas, the sick poor of the Isle of Man are likely to benefit materially. His trustees contemplate spending £7,000 to improve Noble's Isle of Man Hospital, to maintain district nurses, and to bear the cost of sending consumptives to sanatoria.

Chairs for research and teaching of protozoology and helminthology are about to be established at the London School of Tropical Diseases.

A new sanatorium for consumptives has been opened in Toronto, Canada, where patients are received free when unable to pay. It is maintained by Government grant, civic grant, and private contributions.

We regret to record the death of Deputy-Inspector-General of Hospitals and Fleets George Andrew Campbell, M.D., R.N. (retired). He took his medical degree at Kingston, Canada, in 1859, and served at the bombardment of Alexandria and in Suakin, and was retired as Deputy-Inspector-General in 1891.

The late Mr. Alexander Russell, of St. Louis, U.S.A., a native of Dunfermline, has bequeathed 10,000 dollars each to the Edinburgh Royal Infirmary and the Dunfermline Cottage Hospital, and 5,000 dollars each to the Royal and Western Infirmaries, Glasgow, and 2,500 dollars to the Dunfermline Invalids' Benevolent Fund.

Dr. A. Hillier, the hon. secretary of the National Association for the Prevention of Tuberculosis, has been lecturing at Finsbury on the subject of consumption and its prevention and cure. The announcements which Dr. Hillier was able to make of the good resulting from stringent measures to prevent the spread of tuberculosis where they have been adopted, should prove so convincing and encouraging as to aid the fight against the disease amongst those who attended the lecture. Dr. Hillier especially laid stress on the experience in New York. Since the date of his lecture Canada has recited an equally happy outcome of careful sanitary measures adopted.

The Hon. Stephen Coleridge is very active in his attempts to injure the large London hospitals just at the present moment. The following letter from Mr. Edgar Speyer, in response to one of Mr. Coleridge's "inquiries" at the National Hospital for the Paralysed and Epileptic, is interesting as exemplifying a very just attitude of defence on the part of those in authority in the hospitals.

As you are already aware, the National Hospital is not a place licensed under the Act for experiments on living animals. I am informed, nevertheless, that your society endeavoured to prevent subscriptions being sent to it on the ground that some members of the medical staff, in their private capacities, are licensed under the Act. The Nervous Diseases Research Fund is an endeavour to provide funds for research into the origin and cure of those diseases. It will be conducted in the hospital under the advice of the medical staff. That being so, your past treatment of the hospital shows that it has nothing to expect from your society in the way of support. As this removes the only *locus standi* you might otherwise have to interfere, I do not think it necessary to enter further into the subject of your letter.

